# Association between appearance schemas and personality traits

**DOI:** 10.1192/j.eurpsy.2021.1987

**Published:** 2021-08-13

**Authors:** B. Maia, M. Marques, F. Carvalho

**Affiliations:** 1 Faculty Of Philosophy And Social Sciences, Centre For Philosophical And Humanistic Studies, Portugal, Universidade Católica Portuguesa, Braga, Portugal; 2 Coimbra Hospital And University Centre, Portugal, Coimbra Hospital and University Centre, Coimbra, Portugal; 3 Institute Of Psychological Medicine, Faculty Of Medicine, University of Coimbra, coimbra, Portugal; 4 Espaço Psicológico – Consultório De Psicologia, Coimbra, Portugal

**Keywords:** University Students, appearance schemas, Personality

## Abstract

**Introduction:**

Personality traits play are related to many forms of psychological distress, such as body dissatisfaction.

**Objectives:**

To explore the associations between appearance schemas and personality traits.

**Methods:**

494 university students (80.2% females; 99.2% single), with a mean age of 20.17 years old (SD=1.77; range:18-20), filled in the Appearance Schemas Inventory-Revised, the NEO-Personality Inventory, and the Composite Multidimensional Perfectionism Scale.

**Results:**

A significant difference was found in Self-evaluation Salience scores by sex [females (M=37.99,SD=7.82); males (M=35.36,SD=6.60);t(489)=-3.052,p=.002]. Having conducted correlations separately, by sex, Self-Evaluation Salience was correlated with Concern Over Mistakes (r=.27), Doubts about Actions (r=.35), and Socially-Prescribed Perfectionism (r=.23). For females, Self-evaluation Salience was correlated with Concern Over Mistakes (r=.34), Personal Standards (r=.25), Doubts about Actions (r=.33), Parental Expectations (r=.24), Parental Criticism (r=.24), Organization (r=.11), Socially-Prescribed Perfectionism (r=.31), Self-Oriented Perfectionism (r=.32), and Neuroticism (r=.33). Concerning Motivational Salience, in the total sample (not separately, by sex), this scale/dimension significantly correlated with Conscientiousness (r=. 18), Personal Standards (r=.23), Socially-Prescribed Perfectionism (r=. 10), and Self-Oriented Perfectionism (r=.29).

**Conclusions:**

Females seem to value more their self-appearance and, in females, the salience of appearance in life seems to be associated with maladaptive-perfectionism, as well as with adaptive-perfectionism. In males, the salience of appearance was only related with adaptive perfectionism. Males seem more concerned with their own standards, while for females other´s standards are also relevant. In females the level of salience of appearance in life seems to relate to the experience of feelings, such as anxiety/depression (neuroticism). The motivation to improve appearance seemed to be particularly related, in both sex, to adaptive perfectionism.

**Disclosure:**

No significant relationships.

